# Possible Application of Melatonin in Long COVID

**DOI:** 10.3390/biom12111646

**Published:** 2022-11-07

**Authors:** Daniel P. Cardinali, Gregory M. Brown, Seithikurippu R. Pandi-Perumal

**Affiliations:** 1 Faculty of Medical Sciences, Pontificia Universidad Católica Argentina, Buenos Aires C1107AAZ, Argentina; 2 Centre for Addiction and Mental Health, Department of Psychiatry, University of Toronto, Toronto, ON M5T 1R8, Canada; 3 Saveetha Medical College and Hospitals, Saveetha Institute of Medical and Technical Sciences, Saveetha University, Chennai 600077, India

**Keywords:** brain fog, COVID-19, fibromyalgia, long COVID, melatonin, minimal cognitive impairment, myalgic encephalomyelitis/chronic fatigue syndrome, SARS-CoV-2 virus

## Abstract

Clinical sequelae and symptoms for a considerable number of COVID-19 patients can linger for months beyond the acute stage of SARS-CoV-2 infection, “long COVID”. Among the long-term consequences of SARS-CoV-2 infection, cognitive issues (especially memory loss or “brain fog”), chronic fatigue, myalgia, and muscular weakness resembling myalgic encephalomyelitis/chronic fatigue syndrome (ME/CFS) are of importance. Melatonin may be particularly effective at reducing the signs and symptoms of SARS-CoV-2 infection due to its functions as an antioxidant, anti-inflammatory, and immuno-modulatory agent. Melatonin is also a chronobiotic medication effective in treating delirium and restoring the circadian imbalance seen in COVID patients in the intensive care unit. Additionally, as a cytoprotector, melatonin aids in the prevention of several COVID-19 comorbidities, including diabetes, metabolic syndrome, and ischemic and non-ischemic cardiovascular diseases. This narrative review discusses the application of melatonin as a neuroprotective agent to control cognitive deterioration (“brain fog”) and pain in the ME/CFS syndrome-like documented in long COVID. Further studies on the therapeutic use of melatonin in the neurological sequelae of SARS-CoV-2 infection are warranted.

## 1. Introduction

The COVID-19 pandemic’s repercussions are catastrophic. Over 619 million persons worldwide have had proven infection with the SARS-CoV-2 virus as of 5 October 2022, and over 6.5 million have died [1]. Although the SARS-CoV-2 virus has a higher preference for lung tissue, clinical and experimental research have also revealed its strong affinity for the central nervous system [2]. Associated with SARS-CoV-2 infection, neurological symptoms such as memory loss, lack of concentration, myalgia, anosmia, cephalea, and dizziness are commonly reported in COVID-19 patients, as well as isolated cases of epileptic seizures, encephalitis, stroke, and rhabdomyolysis [3]. The long-term consequences of SARS-CoV-2 infection include cognitive issues (especially memory loss or “brain fog”), chronic fatigue, myalgia, muscular weakness, persistent fever, and shortness of breath on exertion [3].

Breakthrough SARS-CoV-2 infection may occur in vaccinated individuals. A study including 33,940 individuals with breakthrough SARS-CoV-2 infection and followed for up to 6 months after infection indicated a higher risk of death and incident post-acute sequelae, including deteriorated mental health, musculoskeletal, and neurologic disorders [4,5,6,7]. As compared to people with SARS-CoV-2 infection who had not previously been vaccinated, patients with breakthrough SARS-CoV-2 infection had decreased odds of mortality and post-acute incident sequelae [4,5,6,7]. Hence, immunization before infection provides only limited protection in the post-acute phase of COVID-19 illness, and a single mitigation technique may not diminish the long-term health implications of SARS-CoV-2 infection optimally [4,5,6,7].

In a 2-year retrospective cohort analysis of neurological and mental sequelae, 1,284,437 patients with SARS-CoV-2 infection were matched with controls who had a different respiratory condition [8]. While the increased incidence of mood and anxiety disorders was transient, the increased risk of cognitive deficit, dementia, psychotic disorder, or seizures persisted throughout. Neurological and psychiatric outcomes were similar during the delta and omicron waves suggesting that pressure on the healthcare system might continue even with variants that are less severe in other aspects [8]. Overall, the findings highlight the need to optimize options other than vaccinations for the primary prevention of SARS-CoV-2 infection.

Melatonin, an effective chronobiotic/cytoprotective chemical, has been recommended as a therapy since the onset of the COVID-19 pandemic [9,10,11,12,13]. The justification for its use not only derives from its multiple beneficial effects in experimental and clinical studies related to SARS-CoV-2 infection but is also due to its high-security profile. Melatonin (a) impairs SARS-CoV-2 infection; (b) is an effective antioxidant/anti-inflammatory/immunoregulatory compound; (c) restores and maintains circadian rhythmicity; (d) is effective to treat COVID-19 comorbidities such as metabolic syndrome, diabetes mellitus, and cardiovascular diseases; (e) is an effective neuroprotector in SARS-CoV-2 patients; (f) potentiates anti- SARS-CoV-2 vaccines (see Ref. [14]). Recently Jarrot et al. put forth the hypothesis that melatonin, an agent that activates the intracellular transcription factor nuclear factor erythroid-derived 2-like 2, enhancing expression of glutathione-synthesizing enzymes, should be considered in the treatment of long COVID [15]. In this review, we discuss the potential use of melatonin to treat brain fog and myalgic encephalomyelitis/chronic fatigue syndrome (ME/CFS) in long COVID.

## 2. Brain Fog in Long COVID

The term “brain fog”, also known as cognitive dysfunction, comprises deficits in attention, short-term and working memory, verbal and non-verbal learning, mathematic problem-solving, and processing speed, focusing on a specific topic [16]. Brain fog is not always related to an underlying condition and may be caused by chronic stress, poor sleep, hormonal changes such as pregnancy or menopause, poor nutrition, chemotherapy, or viral infection, remarkably SARS-CoV-2 [17].

According to research conducted by Northwestern University’s Neuro-COVID-19 Clinic, brain fog was the most common (>6 weeks) neurologic symptom among patients (81%) who did not have a history of acute sickness, hypoxia, or respiratory compromise [18]. Fatigue was the most frequently reported complaint (58%) in a systematic review assessing the prevalence of symptoms in long COVID [19].

A systematic review assessing the prevalence of symptoms of long COVID has been published [19]. For chronic trouble concentrating and attention problems, the effects on cognition were found to be 31% and 27%, respectively. Short-term memory (32%) and attention (27%) abnormalities were also seen in patients with brain fog. Neuroinflammation resulting in hypometabolic lesions has been hypothesized as one cause of chronic dysfunction following moderate COVID disease [20].

A significant cognitive deficit was detected in a study on 84,285 individuals with biologically confirmed COVID-19 infection who completed a Great British Intelligence Test [21]. The sample included people who had recovered, many of them no longer reporting symptoms and the magnitude of the detected deficiencies was similar to the average 10-year loss in performance observed worldwide at ages 20–70 years [21]. Brain fog, or disorientation, forgetfulness, inability to focus, exhaustion, and poor mental energy, is, therefore, an important developing consequence of COVID-19 infection. Essentially, brain fog can be considered an example of minimal cognitive impairment (MCI) [22].

## 3. Magic Encephalomyelitis/Chronic Fatigue Syndrome in Long COVID

Myalgic encephalomyelitis/chronic fatigue syndrome (ME/CFS) is a multi-systemic condition with devastating and often lifelong symptoms characterized by persistent fatigue, intolerance to physical exercise, cognitive problems, sleep disruption, and underlying autonomic dysfunction [23]. ME/CFS is associated with an oxidative and nitrosative stress state, mitochondrial dysfunction together with dysregulated bioenergetics condition, a proinflammatory state, and disruption of the gut mucosal barrier [24]. Disruption of circadian rhythmicity occurs in ME/CFS as indicated by the disrupted rhythms in sleep, activity, and cognition with concomitant insomnia, energy disturbances, cognition problems, depression, and autonomic dysfunction [25]. A key role of disrupted circadian transforming growth factor-β (TGF-β) signaling in ME/CFS was proposed in this respect [25].

Initially, post-COVID-19 follow-up studies included patients with the most severe infection and who spent, on average, 2 weeks in the intensive care unit [26]. These follow-up data documented the occurrence of severe consequences in many patients. One of these studies reported that 45.2% of COVID-19 patients met specific ME/CFS criteria 6 months later [27]. Post-exertional malaise, one of the major ME/CFS symptoms, was observed in 56.8% of a sample of 3762 COVID-19 patients across 56 countries [28]. Thus, the post-COVID-19 condition is presently recognized as having a considerable overlap of symptoms with ME/CFS.

## 4. Mechanism of Action of Melatonin Relevant to Long COVID Treatment

Melatonin is an ancient molecule. This methoxyindole is found in all forms of life that express aerobic respiration; melatonin’s primary function is cytoprotection, displaying anti-inflammatory, antioxidant, and immunostimulant effects [29,30] which together endow it with highly potent neuroprotective properties [31]. The anti-inflammatory action of melatonin involves a variety of mechanisms [32]. One of them is Sirtuin-1 induction, which decreases the polarization of macrophages toward a proinflammatory profile [33]. Suppression of nuclear factor (NF)-κB activation [34,35] and stimulation of nuclear erythroid 2-related factor 2 are also detected after exposure to melatonin [36]. Melatonin reduces proinflammatory cytokines (tumor necrosis (TN)F-α, interleukin (IL)-1β, IL-6, and IL-8) and increases anti-inflammatory cytokines such as IL-10 [33,37].

The antioxidant and scavenging effects of melatonin on free radicals in both the cytoplasm and the cell nucleus are mainly independent of receptors [38]. To fulfill this, melatonin not only acts as a free radical scavenger but also gives rise to a cascade of molecules with high antioxidant activity. It also acts as an indirect antioxidant, enhancing the production of antioxidant enzymes while inhibiting that of prooxidant enzymes [39]. In addition, some antiapoptotic and cytoprotective effects are seen under ischemia, presumably due to melatonin’s stabilizing activity of the mitochondrial membrane [40].

A distinguishing hallmark of viral infection is the shift of cellular metabolism from the oxidative phosphorylation pattern taking place in the mitochondria to glycolysis occurring mainly in the cytoplasm (Warburg’s effect) [41]. The main phenomenon responsible for the change in the oxidation of mitochondrial glucose is the positive regulation of cytoplasmic pyruvate, which is often accompanied by the increase in the hypoxia-inducible factor-1α (HIF-1α), and of NF-κB and other transcription factors promoting inflammation [42]. Because of this, M2 anti-inflammatory macrophages in COVID-19 patients are converted into M1 proinflammatory cells, therefore triggering a cytokine storm. Thus, melatonin can reduce the damage resulting from sepsis mediated by COVID-19 through different mechanisms, i.e., by reversing the Warburg-type metabolism and transforming proinflammatory M1 macrophages into anti-inflammatory M2 macrophages [43], by mitigating the production of HIF-1α [44], by suppressing NF-κB [45], and by inhibiting NLRP3 inflammasome [46]. Circulating secreted phospholipase-A2 (Group IIA) correlated with the severity of COVID-19 disease [47]; hence, cyclooxygenase inhibition by melatonin [48,49] is another potential mechanism by which the methoxyindole may inhibit viral infection.

As shown by several meta-analyses, the chronobiotic/hypnotic properties of melatonin are useful in patients with sleep disorders by synchronizing the circadian apparatus, decreasing sleep onset latency, and increasing total sleep time [50,51,52]. A significant role of melatonin treatment in adult insomnia was the conclusion of several recent expert consensus reports [53,54,55,56]. In addition, melatonin reduces the need for sedation in ICU patients [57,58,59,60,61,62]. These chronobiotic/hypnotic effects of melatonin are obtained at a daily dose range of 2–10 mg [63].

It may well be true that higher doses of melatonin would be more beneficial in the COVID pandemic condition. For example, in a retrospective cross-sectional study of a closed population of 110 old adult patients treated with a mean melatonin daily dose of 46 mg for at least 12 months prior to the availability of COVID-19 vaccination, there was no death in the face of a lethality rate of 10.5% in the local population of elders suffering acute COVID-19 disease [64]. Indeed, animal studies support the use of high doses of melatonin to prevent infection in murine COVID-19 models [65]. From several animal studies, the human equivalent dose HED) of melatonin was calculated by allometry for a 75 kg adult [46]. Allometry is commonly employed for determining initial doses used in Phase I human clinical drug trials [66].

(a) Melatonin and brain fog

**Figure 1:**
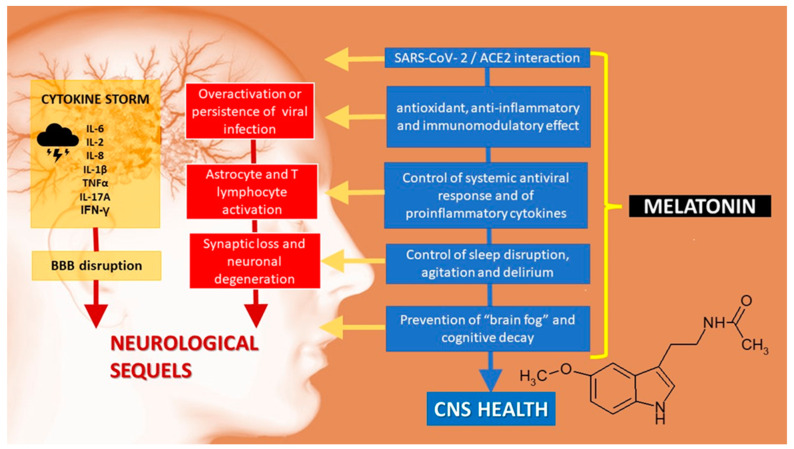
Central nervous system sequelae of SARS-CoV-2 infection. ACE2: angiotensin-converting enzyme 2. BBB: blood–brain barrier.

As stated above, the deficits in attention, memory, verbal processing, and problem-solving seen in patients complaining of brain fog resemble MCI, the initial phase of Alzheimer’s disease (AD) [22]. The underlying neuroinflammation in this condition (Figure 1) could be effectively controlled by melatonin, as shown by studies in cell lines linked to AD, in which melatonin reverses abnormalities in the Wnt/β-catenin, insulin, and Notch signaling pathways, proteostasis disruption and abnormal autophagic integrity (reviewed in Refs. [67,68,69,70,71]).

In transgenic models of AD, melatonin regulates amyloid-β (Aβ) metabolism beginning with the initial phases of the pathological process (see Ref. [31]). From these studies, the HED of melatonin for a 75 kg adult was 2 to 3 orders of magnitude greater than those usually employed in humans. The exact mechanism by which melatonin inhibits the production of Aβ is unknown. Via structural melatonin features independent of its antioxidant capabilities [72], melatonin interacts with Aβ40 and Aβ42, thus inhibiting progressive -sheet and/or amyloid fibrils and facilitating peptide clearance by increasing proteolytic degradation.

Aβ-induced neurotoxicity and cell death involve oxidative stress, and melatonin effectively protects cells against it in vitro [73] and in vivo [74,75]. Melatonin was found to protect against Aβ toxicity, particularly at the mitochondrial level. Melatonin effectively inhibits tau hyperphosphorylation in N2a and SH-SY5Y neuroblastoma cells by influencing protein kinases and phosphatases [76,77].

Melatonin treatment of AD transgenic mice increases Aβ glymphatic clearance [78,79]. Relevant to this, melatonin is known to preserve slow-wave sleep in patients [80], a phase in which the glymphatic elimination of Aβ peptides increases considerably [81]. Thus, the correction by melatonin of sleep disruption can contribute to counteracting the failure of Aβ clearance found in AD.

Epidemiological research suggests that anti-inflammatory medication use in AD may be beneficial due to activated microglia’s decreased secretion of proinflammatory cytokines [82]. In this respect, melatonin is very effective in attenuating the microglial production of proinflammatory cytokines induced by Aβ, NF kB, or nitric oxide [83].

The effectiveness of melatonin therapy in improving sleep in demented patients is supported by two meta-analyses [84,85]. In addition, the administration of melatonin in the initial stages of cognitive decline consistently improves sleep and cognitive performance (see Ref. [31]). In one of our laboratories, we conducted a retrospective analysis of MCI patients who had received a daily dose of 3–24 mg of melatonin along with their usual medication. Compared to the untreated group, melatonin-treated patients significantly improved cognitive performance, Beck Depression Inventory, and quality of sleep/wake rhythm [86,87]. In a study on 40 MCI patients treated with melatonin at a daily dose of 0.15 mg/kg for 6 months, the hippocampal volume and lamina cribrosa thickness decreased significantly as compared with 39 MCI patients receiving placebo [88]. On the other hand, the cerebrospinal fluid T-tau level of the melatonin-treated group was significantly lower compared with the untreated group. A lower Mini Mental State Examination score, a smaller hippocampus volume, and upregulated level of tau protein were associated with significantly thinner lamina cribrosa in MCI patients, all effects counteracted by melatonin treatment [88]. In a meta-analysis of 22 randomized controlled trials to assess the neurocognitive effects of melatonin treatment in healthy adults and individuals with AD disease and insomnia, AD patients receiving >12 weeks of melatonin treatment (2.5–10 mg daily) improved MMSE score, particularly in the mild stage of AD [89]. Therefore, melatonin treatment could be effective in the early stages of neurodegenerative diseases, such as brain fog, in long COVID patients. Unfortunately, very little information is available on melatonin efficacy in COVID therapy, and none has been related to long COVID brain fog syndrome.

(b) Melatonin and ME/CFS

**Figure 2:**
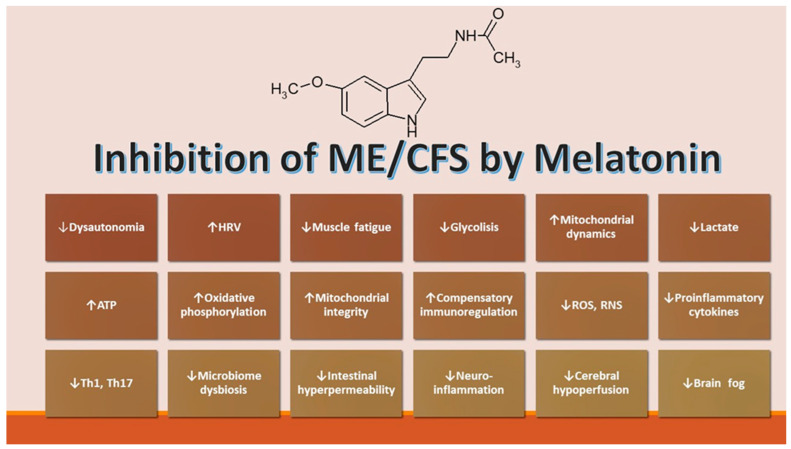
Putative activity of melatonin in ME/CFS. HRV: heart rate variability.

The beneficial effects of melatonin on fibromyalgia (associated commonly with ME/CFS) were first described in one of our laboratories [90]. Since then, several studies have confirmed the initial findings (for a summary, see ref. [91]). A common pathogenic mechanism is suggested by the similarities among ME/CFS, fibromyalgia, and post-COVID syndrome. The multiplicity of pathophysiological abnormalities in ME/CFS patients opens the possibility of numerous potential therapeutic targets [24]. The several abnormalities described comprise increased oxidative stress, mitochondrial dysfunction, dysregulated bioenergetics, a proinflammatory state, the disruption of gut mucosal barriers, and autonomic nervous system disturbances related to autoimmunity [92] (Figure 2). The possible therapeutic options targeting these pathways include melatonin, coenzyme Q10, curcumin, molecular hydrogen, and N-acetylcysteine [24]. Among them, melatonin is the only compound that addresses all mentioned potential targets [24].

## 5. Conclusions

Considering the quantity of scientific/medical studies that have suggested melatonin use in the COVID-19 pandemic, the inability of melatonin to garner attention from public health authorities or the pharmaceutical industry is disheartening. More than 190 papers on pubmed.gov (accessed on 9 October 2022) have examined the use of melatonin as a safe and potentially effective therapy for the COVID-19 pandemic since its inception [93]. This might be due to several factors, including the fact that no influential organization has promoted its therapeutic use for this condition. Melatonin is non-patentable and cheap; therefore, the pharmaceutical business has little motive to encourage its usage. Meanwhile, several potentially harmful and costly medications have been repackaged as therapies for this disease [94].

In critical situations, such as an Ebola outbreak or the COVID-19 pandemic, it is ethical to use all accessible and safe medicines, even if their usefulness has not been fully demonstrated, especially if the therapy has no major adverse side effects. From an analysis of 27 publications that were surveyed on the ability of drugs to successfully treat COVID-19, it was concluded that melatonin is at least twice as effective as remdesivir or tocilizumab in reducing the inflammatory markers of a coronavirus 2019 infection [94]. Given the substantial number of deaths caused by SARS-CoV-2 infections throughout the world, it seems to us that it is immoral to not take advantage of any such safe therapy, especially if the vaccinations become less effective as the virus continues to evolve. At the very least, well-controlled and powered clinical trials are essential to further establish the current evidence that melatonin is safe and efficacious in treating COVID-19 and its sequelae.
